# Intraoperative Nociception Monitoring Using the NoL Index: Phase-Specific Assessment of Nociceptive Responses During Spinal Surgery

**DOI:** 10.3390/jcm14248960

**Published:** 2025-12-18

**Authors:** Amran Khalaila, Mahmod Hasan, Yaron Berkovich, Ali Sleiman, Eitan Mangoubi, Michael Grach, Umar Ibrahim, Adva Gutman Tirosh, Daniel Shpigelman, Arsen Shpigelman

**Affiliations:** 1 Department of Anesthesiology, Carmel Medical Center, Haifa 3436212, Israel; 2 Department of Orthopedic Surgery, Carmel Hospital, Haifa 3436212, Israel; 3 Faculty of Medicine, Technion Institute of Technology, Haifa 3200003, Israel; 4 Faculty of Medicine, Lithuanian University of Health Sciences, 44307 Kaunas, Lithuania

**Keywords:** nociception monitoring, NoL index, intraoperative analgesia, surgery, anesthesia depth, surgical phases

## Abstract

**Background**: Quantifying intraoperative nociceptive responses under general anesthesia remains challenging, particularly during complex procedures such as spinal surgery. The Nociception Level (NoL) index is a multiparametric tool designed to reflect the dynamic balance between nociception and analgesia in anesthetized patients. This study aimed to evaluate NoL fluctuations during predefined phases of spinal surgery and assess their relationship to anesthetic administration. **Methods**: This prospective observational study enrolled 44 adult patients undergoing lumbar discectomy, laminectomy, or spinal fusion under remifentanil–propofol anesthesia. Continuous NoL monitoring was performed using the PMD100™ system. Sixteen anatomically and procedurally defined surgical phases were analyzed. The primary outcome was the mean NoL value in each phase. The secondary outcome was the association between NoL values and intraoperative infusion rates of remifentanil and propofol. Repeated-measures ANOVA with Bonferroni correction was used for phase comparisons. **Results**: Mean NoL values remained within the target range (10–25) in most phases. However, significant elevations were observed during pedicle screw insertion (mean 27.9, SD ± 17.7), cage insertion (27.6, SD ± 10.5), and flavectomy (28.0, SD ± 27.0), indicating increased nociceptive burden. The lowest NoL values occurred during skin closure (16.6, SD ± 11.2) and discectomy (18.0, SD ± 2.8). Propofol and remifentanil infusion rates remained within standard clinical ranges but were slightly elevated during high-NoL phases. **Conclusions**: Despite standardized anesthesia, distinct nociceptive peaks were observed during specific stages of spinal surgery. These findings suggest that NoL monitoring may help identify high-nociception phases and guide tailored analgesic strategies. Future randomized trials are warranted to assess whether protocolized NoL-guided anesthesia improves intraoperative management and postoperative outcomes.

## 1. Introduction

Assessing nociceptive responses in anesthetized patients remains a clinical challenge, primarily due to the limitations of traditional pain assessment tools, which rely on patient self-report and are therefore inapplicable during general anesthesia. Common pain scales—such as the Numerical Rating Scale (NRS), Visual Analog Scale (VAS), Verbal Rating Scale (VRS), and Faces Pain Scale (FPS)—are designed for conscious patients and are influenced by cognitive and emotional factors [1,2,3]. In anesthetized individuals, clinicians typically rely on indirect signs of autonomic nervous system activation—such as hypertension or tachycardia—to estimate nociceptive load [4]. However, these physiological parameters are non-specific and may be affected by factors unrelated to pain [4]. 

To address this limitation, Ben-Israel et al. developed the Nociception Level (NoL) Index in 2013. This multiparametric monitoring system derives a single index score (0–100) from five physiological signals: heart rate, heart rate variability (0.15–0.4 Hz), photoplethysmographic pulse wave amplitude, skin conductance level, and the frequency and amplitude of skin conductance fluctuations. These inputs are processed in real time by the PMD-100™ monitor (Medasense Biometrics Ltd., Ramat Gan, Israel) using a proprietary algorithm [5]. The NoL index aims to reflect the dynamic balance between nociception and analgesia during anesthesia, rather than simply quantifying nociception itself [5,6,7].

Although the NoL system has been investigated in several surgical contexts, there is limited data on its performance during spinal surgery [6,7,8,9,10], where nociceptive intensity varies significantly across surgical stages—from initial skin incision to deep bony manipulation [4]. This variability presents a unique opportunity to evaluate whether NoL monitoring can objectively capture intraoperative nociceptive fluctuations. Therefore, this prospective observational study aims to quantify NoL values during distinct, predefined surgical phases in three common spinal procedures: discectomy, laminectomy, and lumbar fusion. Additionally, the study seeks to explore the relationship between intraoperative NoL values and anesthetic/analgesic management during these phases [8,9,10].

Several monitoring tools have been developed to estimate intraoperative nociception, including the Patient State Index (PSI) and the Analgesia Nociception Index (ANI) [11,12,13,14]. However, these systems primarily rely on EEG or heart rate variability signals and may reflect general arousal or depth of anesthesia rather than pure nociceptive load [11,12,13,15]. In contrast, the NoL index integrates multiple autonomic parameters—including photoplethysmography, skin conductance, and heart rate variability—into a composite score (0–100) that more specifically reflects the dynamic balance between nociception and analgesia. A NoL value between 10 and 25 is considered indicative of adequate analgesia, whereas values above this range suggest a suboptimal nociceptive–analgesic balance [5,6]. This study therefore focused on the NoL index to capture fine-grained, real-time nociceptive responses throughout the surgical procedure [5,6,7].

### Clarification of Terminology

Throughout this study, the term *nociception* refers to intraoperative physiological responses to surgical stimuli, typically manifested via sympathetic activation [4]. *Antinociception* denotes pharmacologic intraoperative interventions aimed at suppressing these responses to maintain physiological stability [4]. The term *pain* is reserved for the conscious sensory experience reported by awake patients postoperatively, and *analgesia* refers to medications administered to relieve such pain [3].

While several digital indices have been developed to monitor intraoperative nociception—such as the Analgesia Nociception Index (ANI) and the Surgical Pleth Index (SPI) [12,13,14,15,16] —these technologies are generally considered to reflect parasympathetic tone or plethysmographic variability rather than direct nociceptive responses [12,13,15]. Although they have been used successfully in procedures like lumbar discectomy and laminectomy, their specificity for nociceptive stimuli remains a matter of debate [13,15]. In contrast, the NoL index employs a multiparametric algorithm that integrates multiple sympathetic nervous system markers, providing a potentially more accurate distinction between nociceptive and non-nociceptive physiological changes under general anesthesia [5,6,7].

## 2. Methods

### 2.1. Study Design and Population

This single-center, prospective observational study was conducted over six months as part of a Basic Science Research Program. Institutional Review Board (IRB) approval was obtained, and all participants provided written informed consent prior to enrollment. Patients were evaluated between 1 February 2022, and 30 June 2022.

The study was approved by the Carmel Medical Center Research Ethics Committee (Approval ID: [CMC-0053-21], Date: 30 May 2021).

Eligible participants were adults (≥18 years), classified as ASA physical status I–III, scheduled for elective lumbar discectomy, laminectomy, or spinal fusion surgery. Exclusion criteria included pregnancy, BMI > 35 kg/m^2^, mean arterial pressure < 60 or >120 mmHg, heart rate < 45 or >90 bpm, cognitive impairment, substance use disorder, or concurrent participation in another clinical trial.

These criteria were chosen to minimize sources of physiological variability that could compromise the accuracy of NoL measurements. Obesity (BMI > 35 kg/m^2^), abnormal blood pressure or heart rate values can distort the underlying signals used in calculating the NoL index. Cognitive impairment was excluded to ensure reliable consent and postoperative symptom reporting.

Both opioid-naïve patients and chronic opioid users were included. Patients continued their routine medications preoperatively. Premedication consisted of oral oxazepam (0.2–0.25 mg/kg). No patients declined participation or withdrew from the study.

This was a descriptive observational study, and no a priori sample size calculation was performed. The number of enrolled participants was determined pragmatically based on feasibility and the number of eligible patients presenting during the six-month recruitment period.

The sample was recruited using a non-randomized convenience sampling approach based on patient availability and surgical scheduling.

### 2.2. NoL Monitoring System

Nociceptive responses were monitored intraoperatively using the Nociception Level (NoL) system (PMD100™, Medasense Biometrics Ltd., Ramat Gan, Israel), a noninvasive device that collects five physiological parameters via a finger sensor. These parameters include heart rate, heart rate variability (0.15–0.4 Hz), photoplethysmographic pulse wave amplitude, skin conductance level, and the frequency and amplitude of skin conductance fluctuations. The system calculates a composite NoL index score ranging from 0 to 100. The NoL score reflects the dynamic balance between intraoperative nociception (i.e., sympathetic responses to surgical stimuli) and antinociception (i.e., pharmacologic suppression of those responses) under general anesthesia.

### 2.3. Anesthesia Protocol and Intraoperative Management

General anesthesia was induced with fentanyl (1.5 μg/kg IV) and propofol (1–2 mg/kg IV). Neuromuscular blockade was achieved with rocuronium (0.6 mg/kg IV), followed by endotracheal intubation. Anesthesia depth was monitored using the Bispectral Index (BIS), targeting values between 45 and 55.

Anesthetic maintenance included continuous infusions of remifentanil and propofol. Anesthesia and antinociceptive medication (e.g., intraoperative opioid administration) were delivered according to standard clinical practice and were not guided by NoL values. No protocolized antinociceptive adjustments were made based on intraoperative NoL readings. The monitor was used solely for observational purposes, and any changes in remifentanil or propofol dosing were made at the discretion of the attending anesthesiologist.

Propofol infusion was adjusted independently based on BIS monitoring. Patients were monitored with 5-lead ECG, noninvasive or invasive blood pressure, neuromuscular monitoring (TOF-Cuff), BIS, and NoL. The NoL sensor was placed on a finger contralateral to the blood pressure cuff.

### 2.4. Extubation and Postoperative Care

Neuromuscular blockade was reversed with sugammadex (2 mg/kg IV). Extubation was performed once the train-of-four (TOF) ratio exceeded 0.9 and the patient was responsive to verbal commands. Thirty minutes prior to the end of surgery, all patients received acetaminophen (1 g IV) and morphine (0.1–0.15 mg/kg IV). In the post-anesthesia care unit (PACU), morphine (1–2 mg IV every 5–10 min) was administered as needed until a VAS score below 4 was achieved.

### 2.5. Data Collection and Outcome Measures

A trained observer documented intraoperative NoL trends to ensure correct phase-matching and identification of meaningful fluctuations. While NoL interpretation is relatively straightforward, precise synchronization with surgical events requires clinical expertise.

NoL values were recorded during 16 predefined intraoperative phases selected based on anatomical and procedural significance (see Table 1). For each phase, the mean NoL value and standard deviation were calculated. Concurrent propofol and remifentanil dosages were recorded for each phase.

- **Primary outcome**: mean NoL values across surgical phases
- **Secondary outcome**: relationship between anesthetic dosages and NoL values

There were no missing data. A detailed list of surgical phases and their temporal sequence appears in Table 1 and the Results section.

### 2.6. Statistical Analysis

Statistical analysis was performed using IBM SPSS v24. Categorical variables were summarized as frequencies and percentages. Continuous variables were assessed for normality (Shapiro–Wilk test, histograms). Normally distributed variables were presented as mean ± standard deviation (SD), and non-normally distributed variables as median with interquartile range (IQR).

Differences in NoL values across surgical phases were evaluated using repeated-measures ANOVA, acknowledging that this approach assumes equal variance and phase duration. Given the descriptive design, this method was selected to provide an initial understanding of phase-specific nociceptive trends. Where appropriate, Bonferroni correction was applied for post hoc pairwise comparisons.

All tests were two-tailed, and a *p*-value ≤ 0.05 was considered statistically significant.

### 2.7. Ethical Considerations

All data were anonymized and de-identified. Only the principal investigator had access to the re-identification key. The study was approved by the Institutional Review Board and conducted in compliance with ICH-GCP and Ministry of Health regulations.

## 3. Results

A total of 44 patients were included in the study, comprising 25 men (57%) and 19 women (43%). The mean age was 57 years (SD ± 13.3). The average weight was 81.2 kg (SD ± 13.3), and the mean height was 168.7 cm (SD ± 8.6). Eight participants (18%) reported chronic opioid use prior to surgery. Regarding the surgical procedures performed, 3 patients (7%) underwent discectomy, 17 patients (39%) underwent laminectomy, and 24 patients (55%) underwent spinal fusion. Detailed demographic and clinical characteristics are presented in Table 2.

**Figure 1:**
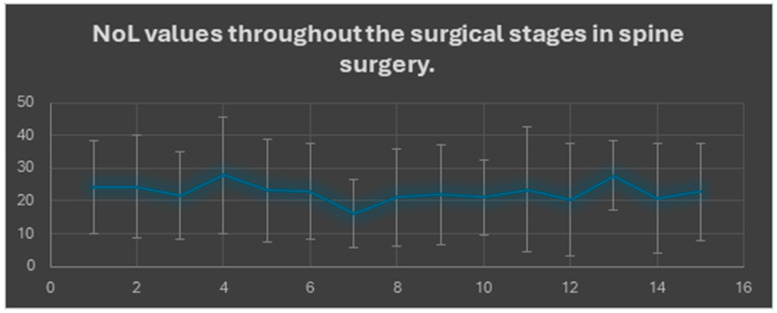
Mean Nociception Level (NoL) values (±SD) across sequential stages of spinal surgery (*n* = 44). Horizontal reference lines indicate the target range (10–25) reflecting optimal intraoperative antinociceptive balance. The *X*-axis represents the various stages of the surgical procedure, while the *Y*-axis shows NoL values (range: 0–100). Data presented are mean values obtained from 44 patients, with standard deviations shown as error bars. Two primary peaks in NoL values are evident at stage 4 (subcutaneous tissue suturing or pedicle screws insertion) and stage 13 (cage insertion or flavectomy), indicating increased sympathetic nervous system activation in response to significant nociceptive stimuli. In contrast, most other stages exhibited lower values, below the threshold of 25, suggesting a milder sympathetic response. These findings demonstrate the potential of the NoL index as an objective measure for evaluating changes in nociceptive stimulus intensity throughout spinal surgeries, highlighting its usefulness as a tool for guiding intraoperative antinociceptive interventions.

The 16 predefined surgical phases were analyzed to evaluate intraoperative nociceptive trends. Analysis of mean Nociception Level (NoL) values across these sequential stages revealed a biphasic pattern. The highest NoL values were observed during pedicle screw insertion (phase 4, mean 27.9, SD ± 17.7) and cage insertion (phase 13, mean 27.6, SD ± 10.5). In contrast, the lowest values were recorded during skin closure (phase 7, mean 16.2, SD ± 10.3). The highest variability was seen in endplate preparation (phase 11, SD ± 19.1), while relatively stable values were noted during discectomy and closure phases. No mean NoL value fell below 10 in any phase. Most phases remained within the target range (10–25), except for phases 4 and 13, where the threshold of 25 was consistently exceeded (Figure 1).

**Figure 2:**
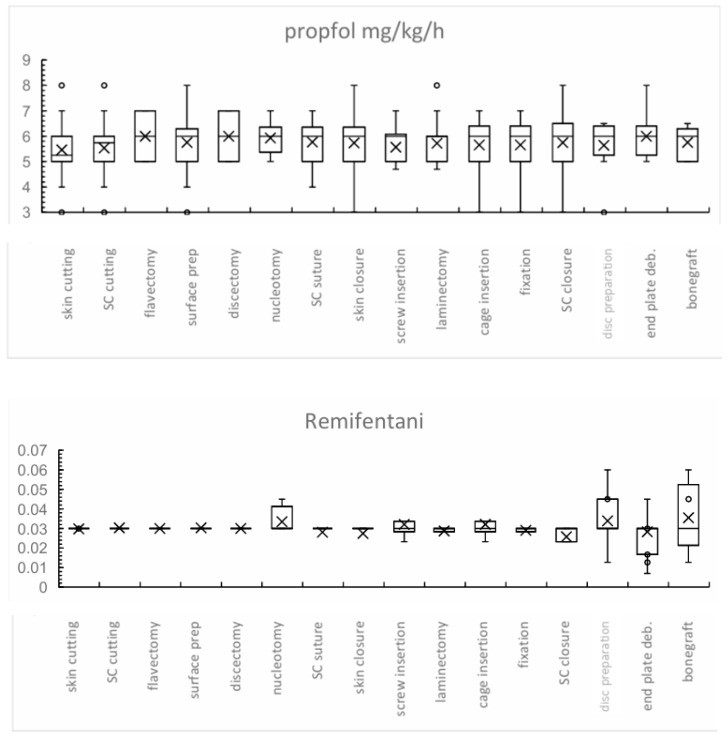
Mean anesthetic dosages of propofol (mg/kg/h) and remifentanil (µg/kg/min) administered across sequential surgical stages. Error bars represent standard deviation. Propofol dosages remained relatively stable, with mean values between 5.4 and 6 mg/kg/h. Remifentanil dosages were also consistent (0.029–0.035 µg/kg/min), with a slight increase observed in advanced surgical stages (e.g., end plate preparation and bone graft insertion), indicating increased sympathetic responses to pronounced nociceptive stimuli. In the boxplots, “X” denotes the mean value, and open circles indicate outliers. Abbreviation: deb. = debridement.

Mean propofol infusion rates ranged from 5.4 to 6.0 mg/kg/h across surgical phases. The lowest rates were noted during initial skin incision (5.4 mg/kg/h, SD ± 1.0), while the highest were observed during discectomy and flavectomy (6.0 mg/kg/h, SD ± 1.4 and ±1.0, respectively). Additional peaks were recorded during endplate debridement and nucleotomy (approximately 5.9 mg/kg/h). Remifentanil infusion rates demonstrated minimal variability, ranging between 29 and 35 ng/kg/min. The lowest rates were recorded during skin closure (29 ng/kg/min, SD ± 0.009), while the highest occurred during bone grafting, disc preparation, and pedicle screw insertion (Figure 2).

**Figure 3:**
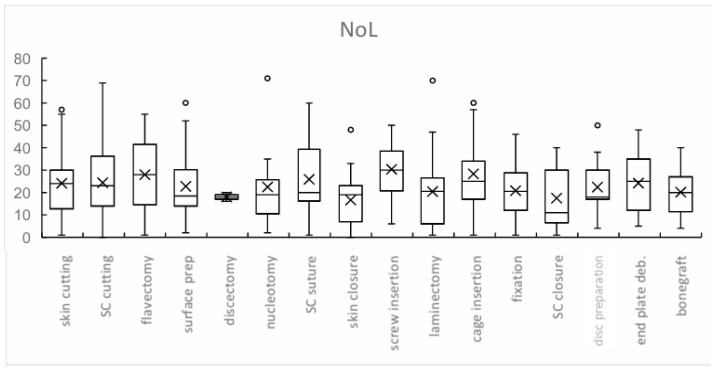
Box plot distribution of Nociception Level (NoL) values across different stages of spinal surgery. Box boundaries represent interquartile range, the central line represents median, ‘X’ represents mean, and whiskers extend to minimum and maximum values (excluding outliers). Mean NoL values (marked by ‘X’) demonstrate variability across stages, with higher values observed during more invasive stages (e.g., screw insertion, cage insertion, and flavectomy), indicating increased sympathetic activation in response to intraoperative nociceptive stimuli. Lower NoL values, suggesting less nociceptive stimulation, were recorded in stages such as discectomy and bone graft insertion. These results highlight the potential utility of NoL monitoring to objectively assess intraoperative nociceptive responses during spinal surgery. In the boxplots, “X” denotes the mean value, and open circles indicate outliers. Abbreviation: deb. = debridement.

When stratifying by procedure type, the highest NoL values were recorded during pedicle screw insertion (mean 30.3, SD ± 13), cage insertion (mean 28.3, SD ± 17), and flavectomy (mean 28.0, SD ± 27). Subcutaneous suturing also showed elevated NoL values (mean 25.9, SD ± 16.4). Phases that approached but did not exceed the 25 threshold included subcutaneous incision (24.4, SD ± 15.7), initial skin incision (24.1, SD ± 14), and tissue debridement (24.2, SD ± 13.1). Intermediate values were observed in disk preparation (22.4, SD ± 14), bone graft placement (20.1, SD ± 12.6), and laminectomy (20.4, SD ± 16.3). The lowest NoL values were found in discectomy (18.0, SD ± 2.8), subcutaneous suturing (17.4, SD ± 14.9), and skin closure (16.6, SD ± 11.2) (Figure 3).

The average timing of surgical phases also varied. Subcutaneous dissection began approximately 3.1 min after skin incision (SD ± 2.5), followed by flavectomy at 7 min (SD ± 2.6), and discectomy at 20 min (SD ± 4.2). Surface preparation was initiated around 25 min (SD ± 27.7), and pedicle screw insertion occurred at approximately 30 min (SD ± 16.2). Laminectomy and tissue debridement followed at 35 and 37 min, respectively. Subcutaneous suturing and nucleotomy were performed at approximately 47 min, while bone graft placement was completed at 67.5 min (SD ± 13.4). Disc preparation and skin closure were typically performed around 74.5 min. Later phases, such as cage insertion (85.8 min, SD ± 39.4), fixation (95.3 min, SD ± 40.3), and final subcutaneous closure (102 min, SD ± 58.4), occurred during the final third of the operation.

## 4. Discussion

This prospective observational study aimed to characterize intraoperative nociceptive responses using the Nociception Level (NoL) index during distinct surgical phases of spinal procedures [5,6,7]. While it is well established that nociceptive intensity varies during surgery, our findings provide structured, phase-specific data illustrating how NoL values fluctuate throughout the course of spinal operations [4]. Notably, several phases—such as pedicle screw insertion and cage placement—consistently produced elevated NoL values, suggesting these stages involve heightened nociceptive load, even under standardized general anesthesia [4].

Our data align with previous studies demonstrating that NoL responds to intraoperative stimuli and reflects activation of the sympathetic nervous system [5,6]. However, unlike subjective tools such as the Visual Analog Scale (VAS), which are limited to conscious patients, the NoL index offers continuous, noninvasive, and objective monitoring of the dynamic balance between nociception and antinociception [1,2,3]. In this context, NoL can provide added insight into intraoperative analgesic adequacy [8,9,10].

In our cohort, NoL peaks consistently emerged during pedicle screw insertion, cage insertion, flavectomy, and subcutaneous tissue suturing. These phases involve manipulation of osseous or richly innervated soft tissues, making the observed sympathetic responses physiologically plausible [4]. Importantly, these peaks persisted despite maintenance of remifentanil and propofol dosages within commonly accepted dosing ranges [17], highlighting the potential limitations of fixed anesthetic regimens and the need for more adaptive, feedback-guided strategies [8,9,10,18].

It is important to interpret these findings with caution. The current study was not designed to assess the clinical efficacy of NoL-guided anesthesia, nor to validate the NoL index as a surrogate for intraoperative pain. While some prior literature supports correlations between NoL and subjective pain measures [9,15], other studies have reported inconsistent associations [19,20]. Factors such as procedural variability, patient characteristics, anesthetic technique, and timing of measurements may account for these discrepancies. Furthermore, comparisons between autonomic indices and subjective pain scores are inherently limited by differences in what they measure: physiological reactivity versus conscious experience [1,3].

This study was observational in design, and NoL monitoring was not used to guide clinical management. Consequently, no control group was included. The purpose was to map intraoperative nociceptive patterns across defined surgical phases, rather than to evaluate the clinical impact of NoL-guided antinociceptive management [8,9,10].

Although our results suggest that NoL may assist in identifying phases of heightened nociception, the directionality between drug administration and NoL response remains unclear in this observational design. For instance, elevated NoL values could reflect delayed or insufficient analgesic dosing, rather than inherent procedural stress. Likewise, the inverse is also possible—persistent peaks despite proactive dosing may indicate limitations of current pharmacologic strategies [8,9,10,18].

To clarify, NoL values were recorded passively and did not influence intraoperative anesthetic or analgesic management. No protocolized adjustments were made based on NoL readings [8,9,10].

Recent investigations continue to expand our understanding of intraoperative nociception monitoring. For example, a large-scale multicenter validation demonstrated the ability of NoL to detect insufficient analgesia across multiple surgical contexts, reinforcing its potential role as a reliable surrogate of autonomic stress responses during anesthesia [9]. Furthermore, a 2022 prospective study suggested that intraoperative NoL fluctuations may predict early postoperative pain trajectories, highlighting the relevance of phase-specific peaks observed in our study [10]. These findings complement machine-learning-based efforts to optimize intraoperative analgesic titration using physiological signals such as NoL, heart rate variability, and skin conductance [5]. Lastly, the integration of NoL into composite monitoring systems has been proposed as a future direction for precision anesthesia, aiming to reduce opioid use and improve recovery metrics [8,9,10,18,21]. While our observational design did not allow causal conclusions, the consistent patterns observed provide a rationale for future interventional studies guided by these evolving frameworks [8,9,10].

### 4.1. Limitations and Strengths

Several limitations must be acknowledged. First, the single-center design and modest sample size (*n* = 44) limit the generalizability of findings. This was a descriptive observational study, and no a priori sample size calculation was performed. The number of participants was determined based on feasibility and recruitment over a six-month period in a single center. Additionally, the inclusion of multiple surgical procedures introduces variability, and the small number of discectomy cases precluded meaningful comparisons by procedure type.

Second, although repeated-measures ANOVA was used to compare NoL values across surgical phases, we acknowledge that this approach assumes uniform phase structure, equal variance, and complete data across all repeated measures. In our study, phase duration varied substantially between patients, and certain phases were not applicable to all procedures, resulting in an unbalanced hierarchical dataset. A mixed-effects model would theoretically better accommodate such inter-subject heterogeneity. However, two practical limitations prevented its application. First, only aggregated phase-level data (mean NoL values per phase) were recorded, rather than patient-level repeated measurements. Second, the primary aim of the study was descriptive—mapping intraoperative nociceptive trends—not modeling predictors or estimating individual effects. Accordingly, repeated-measures ANOVA was selected as a pragmatic tool to visualize general trends, while fully acknowledging its statistical limitations. Future studies should prospectively collect raw patient-level time series data to enable mixed-effects modeling and more precise characterization of intraoperative variability.

Third, no regression analyses were performed to adjust for potential confounders, such as preoperative opioid use or surgical complexity. Fourth, intraoperative dosing was not protocolized based on NoL values, but left to the discretion of anesthesiologists. This introduces the possibility of reverse causation, whereby anesthetic adjustments in response to NoL values may have influenced the observed patterns. Although NoL data were recorded passively and not used in real-time decision-making, future studies should address this potential feedback loop.

Despite these limitations, the study also has strengths. Data collection was systematic, with predefined surgical phases and consistent application of the same validated monitoring device (PMD100™). Furthermore, NoL values were recorded continuously and interpreted by a trained observer, minimizing measurement variability. The consistency of our observed NoL patterns with prior multicenter reports reinforces the potential reproducibility of these findings.

### 4.2. Clinical Implications and Future Directions

**Figure 4:**
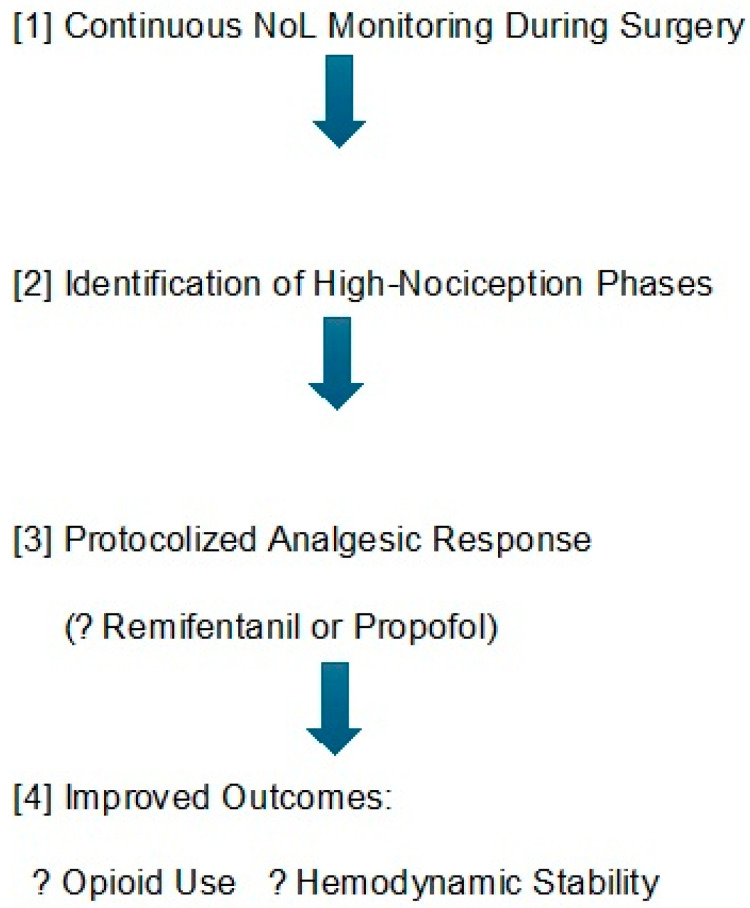
Proposed model for clinical implementation of the NoL index as a feedback-driven intraoperative antinociceptive management tool. Real-time monitoring of nociceptive trends allows identification of high-risk phases (e.g., pedicle screw insertion), which can trigger predefined treatment protocols aimed at improving hemodynamic control and reducing opioid requirements.

These findings are summarized in a proposed model illustrating potential clinical implementation of the NoL index as a feedback-driven antinociceptive tool (Figure 4). While the NoL index offers unique advantages in monitoring nociceptive responses intraoperatively, other indices such as the Analgesia Nociception Index (ANI) and the Patient State Index (PSI) have also been explored. However, those primarily focus on parasympathetic tone or depth of hypnosis, respectively, and may not directly reflect nociceptive stimuli. Our findings reinforce the potential utility of the NoL index in this specific context, although direct head-to-head comparisons remain limited.

The consistent identification of elevated NoL values during distinct surgical phases suggests that standard anesthetic regimens may not adequately address intraoperative nociceptive burden. These insights support the development of anticipatory or phase-specific analgesic strategies to mitigate autonomic surges and hemodynamic instability. Importantly, this study highlights the need for protocolized integration of nociceptive monitoring into anesthetic decision-making, shifting from passive observation toward real-time, feedback-guided titration.

Future randomized controlled trials should compare standard practice with NoL-guided anesthesia protocols incorporating predefined response thresholds. Relevant endpoints may include intraoperative opioid consumption, hemodynamic variability, depth-of-anesthesia metrics, time to extubation, and patient-reported outcomes. Such trials are essential to determine whether NoL monitoring improves perioperative care and can serve as a practical adjunct to modern anesthetic practice, facilitating more adaptive, individualized antinociceptive strategies.

### 4.3. Potential Role of NoL Drop After Nerve Root Decompression

Interestingly, some patients exhibited a marked decline in NoL values following nerve root decompression phases, such as flavectomy or discectomy. This observation may imply that surgical relief of mechanical stress on neural structures leads to a rapid attenuation of sympathetic activation. While our current dataset was not designed to prospectively assess whether such intraoperative NoL declines predict improved postoperative recovery, this hypothesis warrants further investigation. Future studies should explore whether significant NoL reductions following nerve decompression correlate with reduced postoperative pain, decreased analgesic requirements, and lower incidence of chronic neuropathic pain.

Emerging evidence suggests that integrating NoL-guided antinociceptive strategies into intraoperative practice may translate into improved postoperative recovery. Real-time adjustment of antinociceptive dosing based on NoL trends could potentially reduce postoperative pain intensity, decrease opioid requirements, and lower the incidence of opioid-related adverse effects such as nausea and vomiting. By minimizing excessive sympathetic activation during high-nociception phases, NoL-guided management may also contribute to more stable intraoperative hemodynamics. Importantly, more precise control of intraoperative nociceptive burden may reduce the risk of postoperative neuroplastic pain, a well-recognized complication following lumbar discectomy and laminectomy. Future randomized trials are needed to validate these possible clinical benefits.

## 5. Conclusions

In this prospective observational study, the NoL index revealed reproducible fluctuations in intraoperative nociceptive load across different phases of spinal surgery. Elevated NoL values were consistently observed during procedures involving deeper structural manipulation, such as pedicle screw insertion and cage placement, suggesting these moments may present an unmet analgesic need. Although this study does not establish causality or validate NoL as a surrogate for pain, it highlights the index’s potential role in intraoperative assessment.

Further high-quality, multicenter randomized trials are needed to determine whether real-time, protocol-driven NoL-guided analgesia can optimize intraoperative management, reduce opioid exposure, and enhance postoperative recovery.

## Figures and Tables

**Table 1:** Detailed Sequential Surgical Steps by Type of Lumbar Spine Surgery with Associated NoL Measurement Points. Table 1 presents the sequential surgical steps for three types of lumbar spine surgeries—Lumbar Discectomy, Lumbar Laminectomy, and Lumbar Fusion—starting from the skin incision to final wound closure. Each procedure is broken down into its key surgical stages to allow for comparative analysis.

Type of Surgery	Surgery Staging
**Lumbar Discectomy**	Skin cutting, Subcutaneous tissue cutting, Laminotomy/Flavectomy, Disk surface preparation, Disk cutting, Removal of the Disk Nucleus, Subcutaneous suture, Skin closure
**Lumbar Laminectomy**	Skin cutting, Subcutaneous tissue cutting, Laminar surface preparation, Decompression with Partial/Complete Laminectomy, Removal of the Soft Tissue from the Spinal canal, Subcutaneous suture, Skin closure
**Lumbar Fusion**	Skin cutting, Subcutaneous tissue cutting, Laminar surface preparation, Pedicular Screws insertion, Decompression with Partial/Complete Laminectomy, Intervertebral Disk surface, preparation, Removal of the Intervertebral Disk/Nucleus, Intervertebral Disc space preparation, Debridement of the Vertebral Endplates, Bone Graft insertion, Intervertebral Cage insertion, Fixation with Rods, Subcutaneous suture, Skin closure

**Table 2:** Presents the demographic and clinical characteristics of the study group, including gender distribution, age, height, weight, opioid use, and type of lumbar surgery performed.

	**%**	
Sex		
Male	43.18	**19**
Female	56.82	**25**
Mean age (years) ± SD		**13.34 ± 57.00**
Mean weight (kg) ± SD		**13.29 ± 81.26**
Mean height (cm) ± SD		**8.64 ± 168.69**
Opioid Use		
Yes	18	**8**
No	80	**36**
Type of Surgery		
discectomy	6.82	**3**
laminectomy	38.64	**17**
spinal fusion	54.55	**24**

## Data Availability

The datasets generated and analyzed during the current study are not publicly available due to patient privacy and institutional restrictions, but are available from the corresponding author upon reasonable request.
